# Maternal Type-I interferon signaling adversely affects the microglia and the behavior of the offspring accompanied by increased sensitivity to stress

**DOI:** 10.1038/s41380-019-0604-0

**Published:** 2019-11-26

**Authors:** Hila Ben-Yehuda, Orit Matcovitch-Natan, Alexander Kertser, Amit Spinrad, Marco Prinz, Ido Amit, Michal Schwartz

**Affiliations:** 1 Department of Neurobiology, Weizmann Institute of Science, Rehovot, Israel; 2 Department of Immunology, Weizmann Institute of Science, Rehovot, Israel; 3 Institute of Neuropathology, Faculty of Medicine, University of Freiburg, Freiburg, Germany; 4 Signalling Research Centres BIOSS and CIBSS, University of Freiburg, Freiburg, Germany; 5 Center for Basics in NeuroModulation (NeuroModulBasics), Faculty of Medicine, University of Freiburg, Freiburg, Germany

**Keywords:** Autism spectrum disorders, Neuroscience, Schizophrenia, Psychiatric disorders

## Abstract

Viral infection during pregnancy is often associated with neuropsychiatric conditions. In mice, exposure of pregnant dams to the viral mimetic poly(I:C), serves as a model that simulates such pathology in the offspring, through a process known as Maternal Immune Activation (MIA). To investigate the mechanism of such effect, we hypothesized that maternal upregulation of Type-I interferon (IFN-I), as part of the dam’s antiviral response, might contribute to the damage imposed on the offspring. Using mRNA sequencing and flow cytometry analyses we found that poly(I:C) treatment during pregnancy caused reduced expression of genes related to proliferation and cell cycle in the offspring’s microglia relative to controls. This was found to be associated with an IFN-I signature in the embryonic yolk sac, the origin of microglia in development. Neutralizing IFN-I signaling in dams attenuated the effect of MIA on the newborn’s microglia, while systemic maternal administration of IFNβ was sufficient to mimic the effect of poly(I:C), and led to increased vulnerability of offspring’s microglia to subsequent stress. Furthermore, maternal elevation of IFNβ resulted in behavioral manifestations reminiscent of neuropsychiatric disorders. In addition, by adopting a “two-hit” experimental paradigm, we show a higher sensitivity of the offspring to postnatal stress subsequent to the maternal IFNβ elevation, demonstrated by behavioral irregularities. Our results suggest that maternal upregulation of IFN-I, in response to MIA, interferes with the offspring’s programmed microglial developmental cascade, increases their susceptibility to postnatal stress, and leads to behavioral abnormalities.

## Introduction

Viral infections during pregnancy increase the risk to the offspring of developing neuropsychiatric disorders, such as schizophrenia, autism, and bipolar disorders [1–7]. To date, the mechanisms underlying such effects of maternal viral infections, which result in delayed-onset disorders, have not been fully elucidated.

One of the common animal models used to study maternal viral infection outcomes is based on induction of maternal immune activation (MIA) by exposing the dam during pregnancy to the viral mimetic poly(I:C) [8]. MIA includes upregulation of inflammatory cytokines [4, 9–12], such as interleukin-6 (IL-6) that was shown to induce behavioral deficits in the offspring and to elicit changes in gene-expression in their brains [11, 13, 14]. In addition, MIA causes upregulation of interferon beta (IFNβ) and interferon alpha (IFNα), members of the Type-I interferon (IFN-I) family [9, 15, 16], the first line of defense against viral infections in mammals [17, 18]; yet, their possible contribution to the etiology of the offspring’s disorders is still unclear.

An alternative model that was suggested as an explanation for the etiology of neuropsychiatric disorders is the Two-hit model, whereby two “hits” are required for the offspring disorder; a first “hit”, which occurs during prenatal life (such as MIA) disrupts the offspring’s CNS development, thereby increases the vulnerability to a second “hit”, which might occur later in life and would lead to the onset of disorder. In many cases the second “hit” could be an environmental factor such as psychological stress [19–22]. Specifically, early life stress was found in humans as a risk factor that increases vulnerability to neuropsychiatric disorders, and in animal models, maternal separation paradigms are commonly used to study the outcomes of early life stress [23, 24].

Since microglia, the resident myeloid cells of the brain, play a vital role in development, and throughout life in homeostasis and pathology [25–27], they might participate in the processes leading to neuropsychiatric conditions. During development, microglia shape neuronal circuits by elimination of synapses [28–30] and by supporting and regulating neurogenesis [31, 32]. In homeostasis, microglia engage mainly in immune surveillance [33], while during pathology, the microglia become rapidly activated, secrete various cytokines, engage in phagocytic activity, regulate astrocyte pathogenic activity [34], and can promote repair by secreting growth factors [35, 36].

Microglia are regulated by various factors within the central nervous system (CNS) microenvironment [37]. Among them are transforming growth factor beta, which is important for shaping the fate of microglia during development [38] and tightly controls their transcription profile in response to inflammatory signaling [39, 40], methyl-CpG binding protein 2, which regulates microglial response to inflammatory stimuli [41], and interleukin-4, which stimulates microglial proliferation and modifies their inflammatory reaction following acute injury [42]. IFNβ is another important regulator of microglia, and was shown to modify and affect microglial inflammatory responses. As such, IFNβ was shown to reduce microglial-mediated inflammation following spinal cord injury [39] and to promote interleukin-10 production following activation of toll-like receptor (TLR)-3 combined with TLR-2/4/9 [43]. However, prolonged phosphorylation of signal transducer and activator of transcription 1, a protein of the IFN-I cascade, leads to microgliopathy [44, 45], and overexpression of IFNβ in the CNS, as occurs in ageing, induces an ageing-related microglial phenotype that is associated with cognitive impairment [46].

Involvement of immune cells, such as the Th17 population, in promoting an autistic-like phenotype following MIA was shown [4]; nevertheless, the role, if any, of microglia in contributing to the neuropsychiatric pathologies is still subject of debate [47–49]. Several studies that have linked defects in neuronal circuits, such as neuronal excitability and synaptic regulation, to neuropsychiatric disorders [50–54], suggest an active role for microglia in these pathologies [55].

Here, we hypothesized that the maternal elevation of IFN-I, in response to viral infection or viral mimetics, adversely affects the offspring’s microglia and behavior, which are also manifested with increased sensitivity to stressful conditions. We found that following MIA with poly(I:C), the newborn offspring showed reduced microglial proliferation, which was accompanied by an IFN-I signature. Furthermore, systemically blocking maternal IFN-I signaling decreased the effect of poly(I:C), while systemic maternal elevation of IFNβ was sufficient to reduce microglial proliferation in the newborn and to increase the vulnerability of the offspring’s microglia to postnatal stress. In addition, maternal elevation of IFNβ led to behavioral abnormalities in the offspring, and increased their sensitivity to postnatal stress. Overall, this study identifies IFN-I as an important regulator of the fate of microglia in early development, and as a factor that can induce neuropsychiatric-related behavior.

## Materials and methods

### Animals

C57BL/6J, interferon α/β receptor 1 knockout (IFNARKO) [56] and CX3CR1-GFP mice [57] were used. Females were mated at the age of 2–6 months. Newborn (postnatal day 0; P0) pups were treated as described (see below). For behavioral studies the mice were kept in a reverse light–dark cycle (lights off: 9:00–21:00). Animals were supplied by the Animal Breeding Center of the Weizmann Institute of Science. All animals were handled according to the regulations formulated by the Institutional Animal Care and Use Committee.

### Maternal immune activation (MIA)

Timed mating was performed to obtain embryos at defined time points after conception. Females with vaginal plugs were determined as pregnant at embryonic day 0.5 (E0.5). Females were intravenously (i.v.) injected on embryonic day 14.5 (E14.5) with a single dose of 5 mg/kg poly(I:C) (Sigma-Aldrich, Rehovot, Israel) dissolved in PBS, or an equivalent volume of PBS as a control. The dose of poly(I:C) was determined according to Meyer et al. 2008 [58, 59]. The injection volume was 5 ml/kg.

### Maternal treatment with antibodies

On E13.5, pregnant females were i.v. injected with a single dose of 400 µg anti-IFN receptor 1 (αIFNAR; MAR1-5A3 [60], Bio X Cell, NH, USA) or control IgG (IgG1; MOPC-21, Bio X Cell, NH, USA) in 150 µl PBS. After 1 day, on E14.5, the treated females were injected with poly(I:C) or PBS, as described above.

### Maternal treatment with interferon beta (IFNβ)

On E14.5, pregnant females were i.v. injected with a single dose of 22,700 U [61] or 45,400 U recombinant mouse IFNβ (PBL Assay Science, NJ, USA) dissolved in 100 µl vehicle (PBS supplemented with 0.1% FCS), or an equivalent volume of vehicle as a control.

### Maternal treatment with interferon gamma (IFNγ)

On E14.5, pregnant females were i.v. injected with a single dose of 5000 U [62] recombinant mouse IFNγ (R&D systems, MN, USA) dissolved in 100 µl vehicle (PBS supplemented with 0.1% FCS), or an equivalent volume of vehicle as a control.

### Maternal treatment with tumor necrosis factor alpha (TNFα)

On E14.5, pregnant females were i.v. injected with a single dose of 2.7 × 10^5^ U (1 μg) [63] recombinant mouse TNFα (R&D systems, MN, USA) dissolved in 100 µl vehicle (PBS supplemented with 0.1% FCS), or an equivalent volume of vehicle as a control.

### Maternal separation (MS)

The protocol was performed as described [24, 64] with small modifications. In brief, cages were randomly assigned to either MS or nonseparated control groups on the day of birth (P0). In the MS group, pups were separated daily from the dam for a period of 3 consecutive hours randomly during the light phase, from P1 to P14. The dam was first removed from her homecage and placed in a separate cage. All pups were then taken to another room, removed from their homecage and placed separately in small cages filled with clean bedding. Immediately afterwards the dam was returned to her homecage. After the 3 h separation period, the dam was again placed in a separate cage, then all the pups were returned to their homecage, immediately followed by the dam. The control group was left unattended with the dam in the homecage. During the protocol, all the cages were maintained and handled as regularly done in the animal facility.

### Marble burying

The behavioral test was performed to assess repetitive behavior [65, 66]. Twenty glass marbles were placed in an arena sized 25 × 30 cm with 40 cm high walls, filled with clean bedding to a height of 3 cm. The mouse was placed in the arena for 30 min in a dimly-lighted room. Data were recorded using the EthoVision XT 11/14 automated tracking system (Noldus Information Technology). For analysis, the number of buried marbles was counted every 5 min. Marbles that were exposed after being buried were counted as buried. The investigator was blind to the identity of the animals throughout the experiments. Data were analyzed and codes were opened by a member of the team who did not perform the behavioral tests.

### Elevated plus maze

The exploration activity was measured to assess anxiety-related behavior [67]. The mouse was placed in a plus shaped arena elevated 70 cm above the floor, in a lighted room. The sizes of the arms were 30 × 5 cm, while two of them were walled (15 cm high). The mouse was allowed freely to explore the arena for 5 min. Data were recorded using the EthoVision XT 11/14 automated tracking system (Noldus Information Technology). Time spent in the arms and the distance that the animal covered were measured. The investigator was blind to the identity of the animals throughout the experiments. Data were analyzed and codes were opened by a member of the team who did not perform the behavioral tests.

### Social preference

The social behavior of mice was assessed in the three-chamber arena [68]. The arena (60 × 40 cm, 22 cm high walls) is comprised of three chambers of equal size with transparent plexiglass walls. The procedures were conducted in a dimly-lighted room; during the first trial, the mouse was allowed to freely explore the arena for 10 min of habitation, while concurrently, a stranger mouse was placed in a different enclosure, for habituation, inside a cylinder cage (8 × 13 cm) with bars that allow interaction. Subsequently, the tested mouse was guided to the center chamber, while the passages to the side chambers were blocked by opaque partition walls. The stranger mouse and a novel object were placed in each side-chamber of the arena, each inside a cylinder cage. The partition walls were then removed, and the tested mouse was allowed to freely explore the arena and the objects for 10 min. Time spent exploring each object was manually scored using EthoVision tracking system XT 14 (Noldus Information Technology), and percentage exploration of the stranger mouse (social preference) was calculated for each animal, by the formula: Percentage stranger exploration = ((stranger mouse exploration time)/(stranger mouse exploration time + novel object exploration time)) × 100%. The investigator was blind to the identity of the animals throughout the experiments. Data were analyzed and codes were opened by a member of the team who did not perform the behavioral tests.

### Open field arena

The exploration activity was measured to assess anxiety-related behavior [69, 70]. The mouse was placed in an 90 × 90 cm arena located in a lighted room, and allowed to freely explore for 10 min. Data were recorded using the EthoVision XT 14 automated tracking system (Noldus Information Technology). Time spent in arena center and total distance covered by the animal were calculated. The investigator was blind to the identity of the animals throughout the experiments. Data were analyzed and codes were opened by a member of the team who did not perform the behavioral tests.

### Y-maze

Spontaneous alternation behavior was recorded in a Y-maze to assess short-term memory performance [71]. The apparatus was a symmetrical Y-maze; each arm measured 50 × 10 cm, with 40 cm high walls. Mice were placed in the maze, in a dimly-lighted room, and allowed to freely explore for 5 min. Data were recorded using the EthoVision XT 14 automated tracking system (Noldus Information Technology). Arms were arbitrarily labeled A, B, and C, and the sequence of arm entries was used to assess alternation behavior. An alternation was defined as consecutive entries into all three arms. The number of maximum alternations was therefore the total number of arm entries minus two, and the percentage of alternations was calculated as (actual alternations/maximum alternations) × 100. For example, for arms referred to as A, B, and C, if the mouse performed ABCABCABBAB, the number of arm entries would be 11, and the successive alternations: ABC, BCA, CAB, ABC, BCA, and CAB. Therefore, the percentage of alternations would be [6/(11 − 2)] × 100 = 66.7% [72]. The investigator was blind to the identity of the animals throughout the experiments. Data were analyzed and codes were opened by a member of the team who did not perform the behavioral tests.

### Flow cytometry and cell sorting

Newborns (P0), P15 offspring, and dams (negative control group) were transcardially perfused with PBS; brains were dissected and stripped of choroid plexi. Newborn brains were striped of the meninges and cerebellum as well. Single-cell suspensions were achieved using software‐controlled sealed homogenization system (Dispomix®, Medic Tools; Miltenyi) in PBS. For density gradient separation, the pellet was mixed with 40% Percoll and centrifuged at 800 *g* for 20 min at room temperature. For newborn’s microglial Ki67 experiments, single-cell suspensions, following the density gradient separation, were taken for fixation and permeabilization using Cytofix/Cytoperm (BD Biosciences) for 20 min at 4 °C, and washed gently with BD-perm buffer (BD Biosciences). Next, samples were blocked with Fc‐block CD16/32 (BD Biosciences, San Jose, CA) in the presence of 20% donkey serum (10 min at 4 °C), stained and analyzed on a FACS-LSRII cytometer (BD Biosciences) using BD FACSDIVA (BD Biosciences) and FlowJo (FlowJo, LLC) software.

For newborn’s microglia sorting experiments, single-cell suspensions, following the density gradient separation, were blocked with Fc‐block CD16/32, and stained. CD11b^int^CD45^int^ or CD11b^int^CD45^int^CX3CR1^+^ microglia were sorted with SORP-aria (BD Biosciences, San Jose, CA) into 80 μl of Lysis/Binding buffer (Invitrogen).

For intracellular TNFα detection in microglia of P15 pups, single-cell suspensions, following density gradient separation, were incubated with DMEM (Biological Industries) supplemented with 5% FCS, 1 mM l-glutamine, 100 U/ml penicillin, 100 mg/ml streptomycin, and Golgi-stop (1:1000; BD Biosciences) for 3 h at 37 °C, to enable expression of intracellular cytokines, and to prevent their extracellular secretion. Cells were washed, fixed, permeabilized, and stained for surface and intracellular proteins, using Cytofix/Cytoperm kit, according to the manufacturer’s instructions.

The following antibodies were used: BV421-conjugated CD45 (1:150), APC-conjugated CD11b (1:200), PE-conjugated Ki67 (1:150), PE-conjugated CX3CR1 (1:150), APC-conjugated Ly6C (1:150), BV711-conjugated CX3CR1 (1:150), and PE-conjugated TNFα (1:50; all from Biolegend Inc.), and PE-conjugated isotype control Rat IgG1 (1:50; BD Pharmingen).

### Immunofluorescence

Mice were transcardially perfused with PBS before tissue excision and fixation. Tissue processing and immunofluorescence analysis were performed on paraffin-embedded, sectioned (6 μm thick) mouse brains. The following primary antibodies were used: rabbit anti-IFNAR1 (1:50; Abcam), biotinylated-goat anti-GFP (1:150; Abcam), and rabbit anti-IBA1 (1:150; Wako). Secondary antibodies were Cy2-conjugated streptavidin (1:150; Jackson ImmunoResearch), Cy2-conjugated donkey anti-rabbit (1:150; Jackson ImmunoResearch), and Cy3-conjugated donkey anti-rabbit antibodies (1:200; Jackson ImmunoResearch).

A Nikon Eclipse 80i fluorescence microscope was used for microscopic analysis. The fluorescence microscope was equipped with a digital camera (DXM 1200F; Nikon) and with 20× NA 0.50 and 40× NA 0.75 objective lenses (Plan Fluor; Nikon). Recordings were made using acquisition software (NIS-Elements, F3).

Prior to quantification, slices were coded to mask the identity of the experimental groups, and cell intensity was quantified by an observer blinded to the origin of the sample, using Fiji software (ImageJ 1.51w; NIH); the “IntDen” parameter with threshold detection of 94–255 was used to quantify intensity. Five representative images from different depths in the brain were used to calculate the average fluorescence intensity per mouse. Representative images were cropped, merged, and optimized using Photoshop CS6 13.0.1 (Adobe), and were arranged using Illustrator CC 17.0 (Adobe).

### RNA purification and library preparation

mRNA was captured with 12 µl of Oligo(dT) Dynabeads (Life Technologies), washed, and eluted at 70 °C with 10 µl of 10 mM Tris-Cl (pH 7.5). RNA-seq was performed as previously described [73] and DNA libraries were sequenced on an Illumina NextSeq 500 or HiSeq with an average of 4 million aligned reads per sample.

### RNA-sequencing (RNA-seq) processing and analysis

**Fig. 1:**
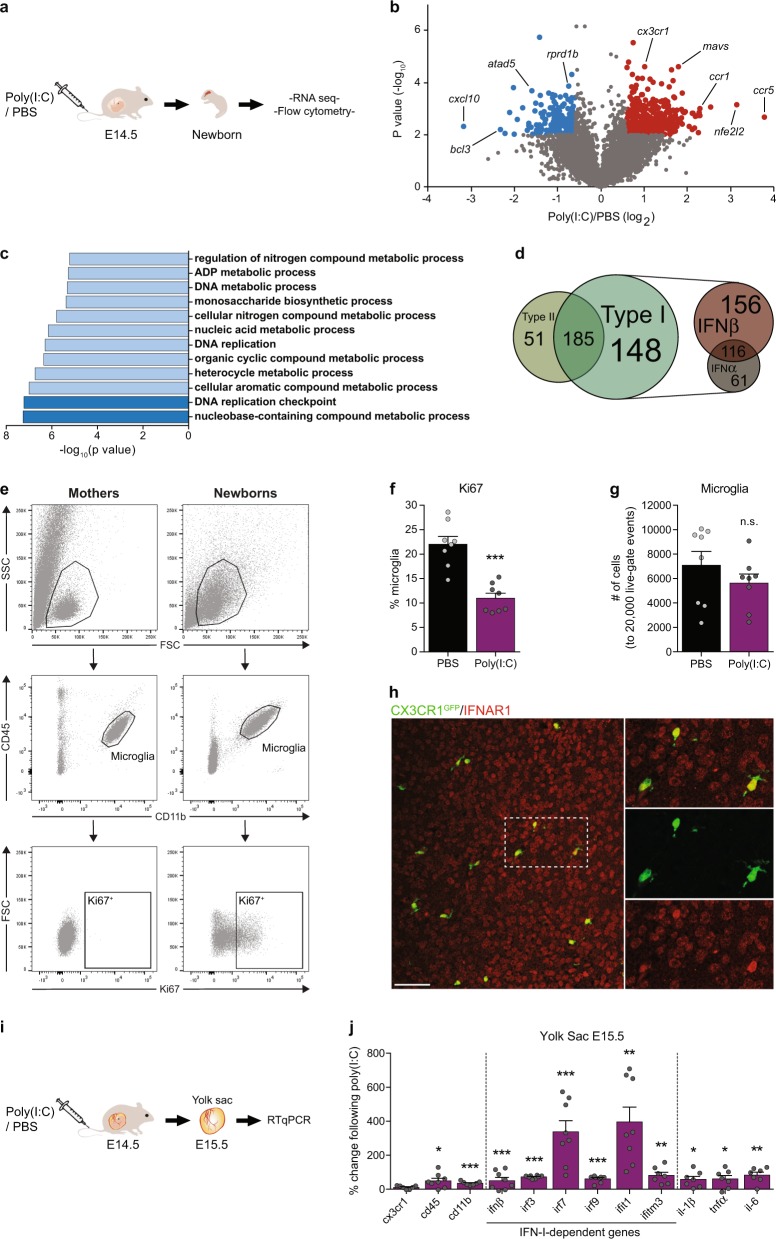
Type-I IFN is associated with reduced offspring’s microglia proliferation and with embryonic yolk sac response following MIA. **a** Schematic presentation of the model used to assess microglial fate: Poly(I:C) (5 mg/kg) or PBS (control) was i.v. injected to pregnant females at embryonic day 14.5 (E14.5), and newborn’s microglia were examined by RNA-seq (published data of Matcovitch-Natan et al. 2016 [74] was analyzed in this study), and by flow cytometry. **b** Volcano plot for the RNA-seq data shows the fold change and significance of genes between microglia of newborn offspring of dams treated with poly(I:C) or PBS [190 genes significantly downregulated (blue) and 404 significantly upregulated (red)]. **c** Gene ontology (GO) analysis [77–80] for RNA-seq shows reduction of terms related to cell cycle and proliferation in microglia derived from newborn progeny of poly(I:C) treated mice. **d** “Interferome” analysis [81] of the RNA-seq results. Three hundred and eighty-four of the affected genes were regulated by Type I and II interferons, while the majority were genes affected specifically by Type-I interferon. IFNβ had the greatest effect, compared with IFNα. **e** Flow cytometry gating strategy for Ki67^+^ microglia. **f** Flow cytometry analyses showing percentage of microglia expressing the proliferation marker, Ki67 (Student’s *t* test: *t*_(two tailed)_ = 5.789*, df* = 14, ***p < 0.001), and **g** normalized number of microglia (to 20,000 live-gate events). **f**, **g** Representative results of one of two independent experiments. Data are presented as means ± s.e.m. *n* = 8 newborns per group. **h** Immunofluorescence staining confirming IFNAR1 expression in CX3CR1-GFP^+^ microglia in the newborn’s brain. Scale bar, 50 µm. **i** Embryonic yolk sacs, the source of microglial progenitors, were excised 1 day following MIA and were analyzed by RTqPCR. **j** RTqPCR results showing percent changes of genes following MIA compared with PBS control. **i**, **j** Representative results of one of two independent experiments. Data are presented as means ± s.e.m. *n* = 7–8 yolk sacs per group. *p < 0.05; **p < 0.01; ***p < 0.001

RNA-seq of newborns following treatment was compared with controls. For the RNA-seq analysis shown in Fig. 1, we analyzed our previous data from Matcovitch-Natan et al. 2016 [74]. The RNA-seq reads were aligned to the mouse reference genome (NCBI 37, mm9) using TopHat v2.0.13 with default parameters [75]. Duplicate reads were filtered if they aligned to the same base and had identical UMIs. Expression levels were calculated and normalized for each sample to the total number of reads using HOMER software (http://homer.salk.edu) with the command “analyzeRepeats.pl rna mm9-d [sample files]-count 3utr-condenseGenes” [76].

Using Microsoft Excel we focused on genes with mean expression above noise (set at 64), that were differentially expressed (with 1.5-fold differential, log fold change = 0.585) between the means of the groups (two-tailed Student’s *t* test, *P* < 0.01).

Gene ontology (GO) associations [77, 78] were determined using GOrilla [79, 80], to which gene symbols of significantly increased or decreased genes (as a target set) and a complete gene list (as a background set) were imported. Interferon related genes were determined using “interferome” [81].

### RTqPCR

Pregnant females at E15.5 were transcardially perfused with PBS, and yolk sacs were extracted. Total RNA was extracted with the RNeasy kit according to the manufacturer’s instructions (Qiagen). RNA was reverse transcribed using the high capacity cDNA reverse transcription kit (Applied Biosystems), amplified using SYBR green I Master Mix (Roche), and detected by StepOnePlus (Applied Biosystems), in duplicates. Results were normalized to the expression of the housekeeping gene, peptidylprolyl isomerase-A (PPIA), and then expressed as percent change relative to the control sample. The following primers were used:

*ppia* forward, 5′-AGCATACAGGTCCTGGCATCTTGT-3, and reverse, 5′-CAAAGACCACATGCTTGCCATCCA-3′;

*cx3cr1* forward, 5′-CTTCCCATCTGCTCAGGAC-3, and reverse, 5′- ACAATGTCGCCCAAATAACAG-3′;

*cd45* forward, 5′-CTATGAGCAATACCAGTTCCTC-3′, and reverse, 5′- GGACGGACACAGTTAGCA-3′;

*cd11b* forward, 5′-TTCAGCAAGCCAGAACCC-3′, and reverse, 5′- CTCAGAATTAGCAGGAAAGATGG-3′;

*ifnβ* forward, 5′-GAATTCGACGTCGGTACCTC-3′, and reverse, 5′-AGGGAACAGCTATGACCATGA-3′;

*irf3* forward, 5′-CTCTGTGGTTCTGCATGGG -3′, and reverse, 5′-TGTAGGAACAACCTTGACCA-3′;

*irf7* forward, 5′-CTTCAGCACTTTCTTCCGAGA-3′, and reverse, 5′-TGTAGTGTGGTGACCCTTGC-3′;

*irf9* forward, 5′-AACCCTCCCTAACCAACCAC-3′, and reverse, 5′- GGTTTACAACGCCATTGGTC-3′;

*ifit1* forward, 5′-CTTTACAGCAACCATGGGAGAG-3′, and reverse, 5′- TCCATGTGAAGTGACATCTCAG-3’;

*ifitm3* forward, 5′-CTGGTCCCTGTTCAATACAC-3′, and reverse, 5′-TTCCGATCCCTAGACTTCAC-3′;

*il-β* forward, 5′-ACCTGTCCTGTGTAATGAAAGAC-3′ and reverse, 5′-TGGGTATTGCTTGGGATCCA-3′.

*tnfα* forward, 5′-CCCTCACACTCAGATCATCTTCT-3′, and reverse, 5′-GCTACGACGTGGGCTACAG- 3′;

*il-6* forward, 5′-TGCAAGAGACTTCCATCCAGTTG-3′ and reverse, 5′-TAAGCCTCCGACTTGTCAAGTGGT-3′.

### Statistics

One or two-tailed Student’s *t* tests were used, as indicated in the figure legends. *p < 0.05; **P < 0.01; ***p < 0.001. Cohen’s *d* was calculated using the conventional formula. Sample sizes were chosen with adequate statistical power based on the literature and past experience, and mice were allocated randomly to experimental groups. Data were excluded only if one of the parameters of the samples was exceeding at least two standard deviations above/below average. Results are presented as mean ± s.e.m. In the graphs, *y*-axis error bars represent s.e.m. Statistical calculations were performed using GraphPad Prism software (GraphPad Software, San Diego, CA).

## Results

### Offspring’s microglia show reduced proliferation and a Type-I interferon signature following maternal immune activation

In our paper by Matcovitch-Natan et al. 2016 [74], we demonstrated an early maturation of newborn’s microglia following MIA. The MIA was induced by injecting pregnant females, on embryonic day 14.5 (E14.5), with poly(I:C) (5 mg/kg) or PBS, as control (the paradigm used is schematically shown in Fig. 1a). Since a viral infection, or administration of the viral mimetic poly(I:C), results in a general upregulation of inflammatory cytokines [4, 9–12], including IFN-I [9, 15, 16], we envisioned that the effect of MIA on the newborn’s microglia might involve IFN-I signaling. To investigate this, we further analyzed RNA-seq data from our previous paper derived from microglia of newborn mice following MIA [74]. We found 594 genes with highly divergent expression between the treated and control groups (190 downregulated and 404 upregulated; Fig. 1b, Supplementary Table 1). GO analysis [77–80] revealed that newborn’s microglia derived from the poly(I:C) group showed lower expression levels of genes related to cell cycle and proliferation (Fig. 1c). In order to test whether the effect of MIA on microglia could be IFN-dependent, we analyzed the RNA-seq results using “Interferome”, the database of IFN-regulated genes [81]. This analysis revealed that approximately two-thirds of the differentially expressed genes (384 genes) are regulated by Type I and II interferons, out of which the majority are genes affected by Type I interferon (Fig. 1d). Specifically, the analysis showed that IFNβ had the greatest effect, compared with IFNα, on newborn’s microglial gene expression following MIA (Fig. 1d).

To confirm the RNA-seq results, we repeated the same MIA procedure and examined the newborn’s microglia for protein expression of the proliferation marker Ki67 by flow cytometry (Fig. 1a, e–g). Microglia from the brains of the dams were used as a negative control, due to their negligible Ki67 expression (Fig. 1e). We found that a significantly lower percentage of microglia derived from offspring of poly(I:C)-treated dams expressed Ki67, as compared with the percentage of microglia derived from offspring of PBS-treated animals (Fig. 1f). Cell quantification showed a trend toward a reduction in the total number of microglia following MIA (Fig. 1g). These results demonstrated that MIA resulted in reduced proliferation of newborn’s microglia.

Next, we assessed whether newborn’s microglia could be affected by IFN-I produced by the dams in response to poly(I:C), as a possible mechanism underlying the effect of MIA (Fig. 1b, d). Accordingly, we checked, using immunofluorescence, whether newborn’s microglia express IFN receptor 1 (IFNAR1). We found that CX3CR1^+^ microglia express IFNAR1 (Fig. 1h), and thus could respond to IFN-I. These results show that MIA downregulates microglial proliferation, and that this effect might involve IFN-I signaling.

### Maternal immune activation elicits a Type-I interferon response in the embryonic yolk sac

The results above led us to hypothesize that if the effect on newborn’s microglia following MIA is mediated through maternal IFN-I, we might detect the IFN-I signature in the yolk sac, the source of microglial progenitors [82, 83], at an early time point after the MIA. To this end, pregnant females were injected on E14.5 with poly(I:C) or PBS, and 24 h later, the embryonic yolk sac was excised and analyzed by RTqPCR (Fig. 1i). We detected increased expression levels of IFN-I-dependent genes [including IFN-β 1 (*ifnβ*), IFN regulatory factor 3 (*irf3*), IFN regulatory factor 7 (*irf7*), IFN regulatory factor 9 (*irf9*), IFN-induced protein with tetratricopeptide repeats 1 (*ifit1*), and IFN-induced transmembrane protein 3 (*ifitm3*)] following MIA (Fig. 1j). Changes in other genes were detected as well, such as NF-kB-dependent inflammatory cytokines [interleukin 1 beta (*il-1β*), tumor necrosis factor alpha (*tnfα*), and interleukin 6 (*il-6*)] [84] and the myeloid markers *cd45* and *cd11b* [85] (Fig. 1j). These results revealed an IFN-I signature in the offspring at an early time point following MIA.

### IFN-I is an important regulator of microglial proliferation

**Fig. 2:**
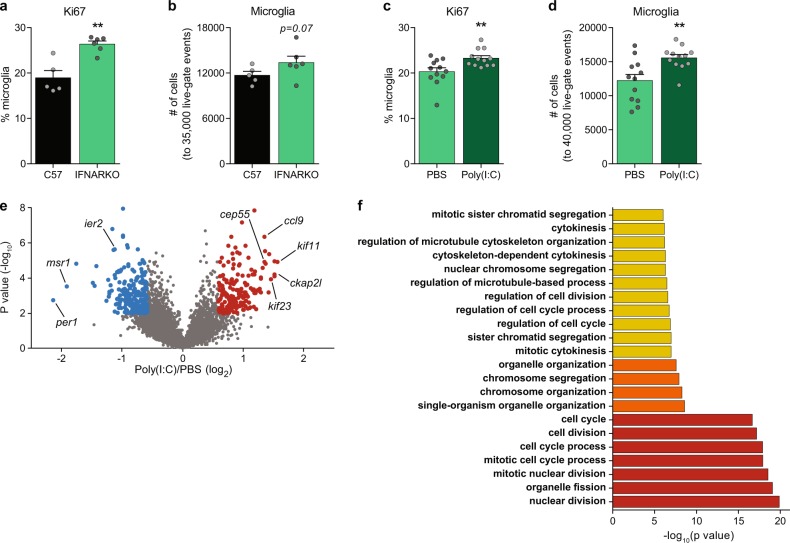
IFN-I regulates microglial proliferation. **a** Flow cytometry analyses showing percentage of C57 and IFNARKO newborn’s microglia expressing the proliferation marker Ki67 (Student’s *t* test: *t*_(two tailed)_ = 4.524*, df* *=* 9, **p = 0.0014*)*, and **b** normalized number of microglia (to 35,000 live-gate events. Student’s *t* test: *t*_(one tailed)_ = 1.603*, df* = 9*, p* = 0.0717). Representative results of one of two independent experiments. Data are presented as means ± s.e.m. *n* = 5–6 newborns per group. **c** Flow cytometry analyses showing percentage of IFNARKO newborn’s microglia expressing the proliferation marker Ki67 (Student’s *t* test: *t*_(two tailed)_ = 2.934*, df* = 22, **p = 0.0077), and **d** normalized number of microglia (to 40,000 live-gate events. Student’s *t* test: *t*_(two tailed)_ = 3.3237*, df* = 22, **p = 0.0038) following MIA. Representative results of one of two independent experiments. Data are presented as means ± s.e.m. *n* = 12 newborns per group. **e** Volcano plot for RNA-seq data shows the fold change and significance of genes of IFNARKO newborn offspring of dams treated with either poly(I:C) or PBS [175 genes significantly upregulated (red) and 230 significantly downregulated (blue)]. **f** Gene ontology (GO) analysis [77–80] of the RNA-seq results showing enrichment of terms related to proliferation and cell cycle following the poly(I:C) treatment

To get a deeper insight into the role of IFN-I during development, we tested whether IFN-I has a homeostatic role in regulating microglial proliferation, using IFNARKO mice [56]. We first compared the basal microglial proliferation in WT and IFNARKO offspring of nontreated dams. Analysis of Ki67 by flow cytometry revealed that a significantly higher percentage of IFNARKO microglia expressed the proliferation marker, as compared with the WT microglia (Fig. 2a). A trend toward an increase in total numbers of microglia was also detected (Fig. 2b). These results suggested that IFN-I participates in the normal regulation of microglial proliferation, and raised the question of whether the newborn’s microglial response to MIA would be different in the absence of IFN-I signaling. To this end, pregnant IFNARKO dams were exposed to poly(I:C) or PBS, and flow cytometry analysis for expression of the proliferation marker Ki67 by newborn’s microglia was performed. We found that following MIA, a significantly higher percentage of IFNARKO newborn’s microglia expressed Ki67, as compared with the PBS-treated group (Fig. 2c). Cell quantification showed a significant increase in total number of microglia, as well (Fig. 2d). RNA-seq of sorted IFNARKO newborn’s microglia from poly(I:C) and PBS groups supported the flow cytometry results (Fig. 2e, f); comparison of poly(I:C) to PBS IFNARKO newborn’s microglia showed highly divergent expression of 405 genes (175 upregulated and 230 downregulated; Fig. 2e, Supplementary Table 2). GO analysis [77–80] showed enrichment of genes related to proliferation and cell cycle in IFNARKO newborn’s microglia following the poly(I:C) treatment, compared with the PBS group (Fig. 2f). The results supported the possibility that IFN-I signaling is involved in regulating newborn’s microglial proliferation in homeostasis. Notably, in the absence of IFN-I signaling, MIA caused an increase in proliferation of newborn’s microglia, and thus elicited a response opposite to that in WT mice. These results further suggest that additional factors, beyond IFN-Ι, participate in regulating microglial proliferation, such as the NF-kB-dependent inflammatory cytokines, IL-1β, TNFα, and IL-6 (Fig. 1j), that might be masked in the presence of IFN-I signaling.

### Maternal IFNβ signaling downregulates newborn’s microglial proliferation

**Fig. 3:**
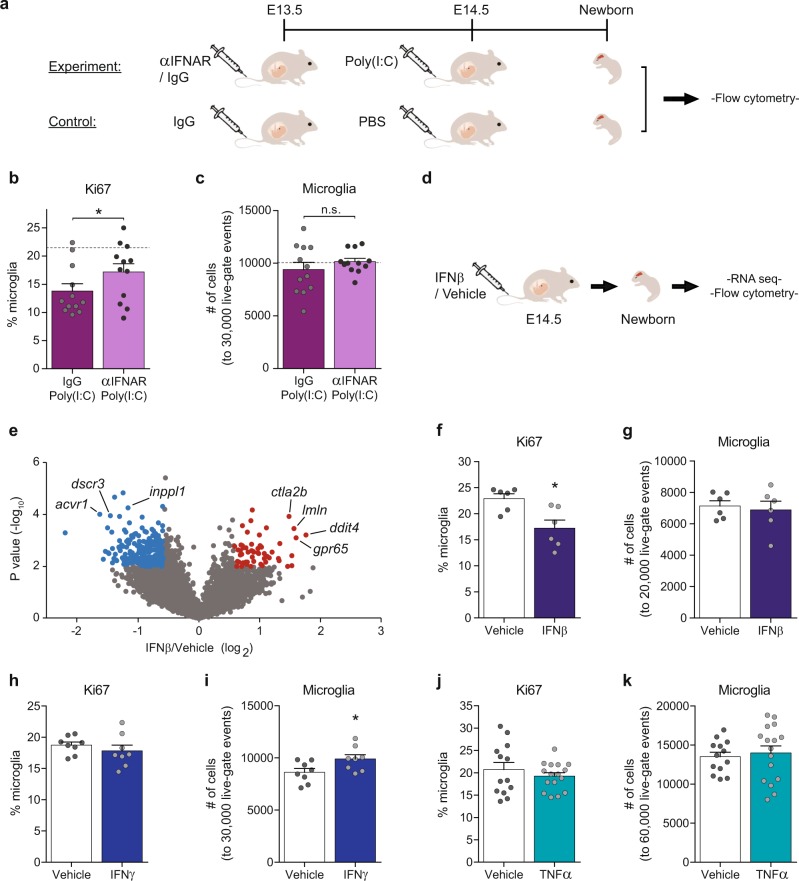
Maternal IFNβ signaling downregulates newborn’s microglial proliferation. **a** Pregnant females were i.v. injected on E13.5 with 400 µg αIFNAR (αIFNAR; MAR1-5A3) or IgG (IgG1; MOPC-21), and 1 day later, on E14.5 with poly(I:C). Females from the control group were injected with IgG on E13.5 and with PBS on E14.5 (IgG-PBS) to determine the basal levels of microglial proliferation and cell numbers. The newborn’s microglia were analyzed by flow cytometry. **b** Flow cytometry analyses showing percentage of microglia expressing the proliferation marker Ki67 (Student’s *t* test: *t*_(one tailed)_ = 1.735*, df* = 22, *p = 0.0484), and **c** normalized number of microglia (to 30,000 live-gate events). Dashed line represents the IgG–PBS control average. Combined results from two different experiments. Data are presented as means ± s.e.m. *n* = 12 newborns per group. **d** Pregnant dams were i.v. injected with IFNβ (22,700 U) or vehicle on E14.5, and newborn’s microglia were examined by RNA-seq and by flow cytometry. **e** Volcano plot for RNA-seq data shows the fold change and significance of gene expression between microglia of newborn offspring of dams treated IFNβ or vehicle [257 significantly downregulated (blue) and 57 significantly upregulated (red)]. **f** Flow cytometry analyses showing percentage of microglia expressing the proliferation marker Ki67 following maternal vehicle and IFNβ treatments (Student’s *t* test: *t*_(two tailed)_ = 3.139*, df* = 10, *p = 0.0105), and **g** normalized number of microglia (to 20,000 live-gate events). Representative results of one of two independent experiments. Data are presented as means ± s.e.m. *n* = 6 newborns per group. **h** Flow cytometry analyses showing percentage of microglia expressing the proliferation marker Ki67 following maternal vehicle and IFNγ treatments, and **i** normalized number of microglia (normalized to 30,000 live-gate events. Student’s *t* test: *t*_(two tailed)_ = 2.339*, df* = 14, *p = 0.0347). Representative results of one of two independent experiments. Data are presented as means ± s.e.m. *n* = 8 newborns per group. **j** Flow cytometry analyses showing percentage of microglia expressing the proliferation marker Ki67 following maternal vehicle and TNFα treatments, and **k** normalized number of microglia (to 60,000 live-gate events). Data are presented as means ± s.e.m. *n* = 13–16 newborns per group

To validate the active involvement of IFN-I, elicited by the dams in response to MIA, in impairing newborn’s microglia, we tested whether blocking IFN-I signaling prior to poly(I:C) treatment would mitigate the effect of MIA on newborn’s microglial proliferation. For this purpose, we used antibodies to block the maternal-IFN-I signaling in WT dams. Specifically, pregnant C57 dams were injected 1 day prior to poly(I:C) treatment [on embryonic day 13.5 (E13.5)], with anti-IFN receptor 1 (αIFNAR; MAR1-5A3 [60], 400 µg) or IgG (IgG1; MOPC-21, 400 µg), to allow efficient blockade before the MIA-induced IFN-I effect on the offspring (Fig. 1i, j). Subsequently, the newborn’s microglia were isolated and tested (Fig. 3a). The control group was injected with IgG on E13.5 and with PBS on E14.5 (IgG–PBS group) to determine the basal levels of microglial proliferation and cell numbers (Fig. 3a). Flow cytometry analysis of newborn’s microglia for the proliferation marker Ki67 showed that maternal treatment with αIFNAR prior to MIA [αIFNAR-Poly(I:C)] diminished the negative effect of MIA on microglial proliferation, as compared with the group that was treated with IgG prior to the poly(I:C) [IgG-Poly(I:C); Fig. 3b]. No changes in the total number of microglia were apparent (Fig. 3c).

These results prompted us to examine whether maternal elevation of IFN-I could, by itself, mimic the effect of MIA on microglial proliferation in the offspring. Since our “interferome” analysis showed that IFNβ had the greatest effect among the IFN-I family members on newborn’s microglial gene expression following MIA (Fig. 1d), we decided to use this cytokine for our subsequent experiments. Pregnant dams were injected at E14.5 with IFNβ (22,700 U) [61] or vehicle, as a control (see Materials and Methods), and the microglia of newborn offspring were examined (Fig. 3d). We first verified the signature of the maternal IFNβ treatment on the microglia of the offspring by RNA-seq. Analysis revealed 57 and 257 genes that exhibited at least 1.5-fold increased or decreased expression, respectively, in offspring of IFNβ-treated dams, compared with vehicle-injected controls (Fig. 3e, Supplementary Table 3). We detected upregulation of various genes related to inflammation, IFN signaling, and the viral response (e.g., *ctla2b, lmln, ddit4,* and *gpr65*). Next, we assessed the proliferation of newborn’s microglia by flow cytometry. A significantly lower percentage of microglia expressed Ki67 following maternal IFNβ injection, as compared with controls (Fig. 3f). No change in the total number of microglia was detected (Fig. 3g). These results demonstrated that the mere maternal elevation of IFNβ was sufficient to induce an effect on the microglia of the offspring, which was manifested by their reduced proliferation.

Because other factors are likely involved in the regulation of the newborn’s microglia (Figs. 1i, j and 2), and in order to determine whether maternal IFNβ uniquely reduces their proliferation, we assessed the effect of additional cytokines. To this end, we injected pregnant dams at E14.5 with interferon gamma (IFNγ; 5000 U [62]), a Type-II interferon member (Fig. 1d), or with TNFα [2.7 × 10^5^ U (1 μg) [63]], an NF-kB-dependent inflammatory cytokine (Fig. 1j). Analyses by flow cytometry reveled that maternal treatment with IFNγ did not elicit a change in percentage of newborn’s microglia expressing Ki67 (Fig. 3h), however, a higher number of microglial cells was observed (Fig. 3i). Analyses of the newborn’s microglia following maternal TNFα treatment, did not show any change in percentage of microglia expressing Ki67, nor in number of microglial cells (Fig. 3j, k). These results suggest that although many factors could be involved in regulation of microglia, IFNβ seems to uniquely reduce their proliferation.

### Elevated IFNβ during pregnancy imposes behavioral abnormalities in the offspring

**Fig. 4:**
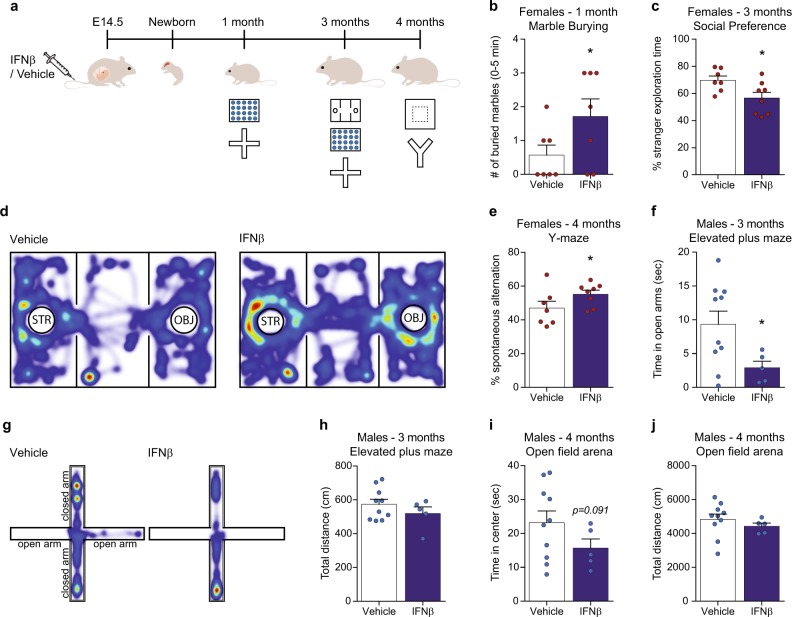
Maternal elevation of IFNβ leads to behavioral abnormalities in offspring. **a** Pregnant females were i.v. injected on E14.5 with IFNβ (45,400 U) or vehicle. At the age of 1 month, the offspring were tested by marble burying and elevated plus maze tests. At the age of 3 months the offspring were tested by social preference, marble burying, and elevated plus maze tests. At the age of 4 months the offspring were tested by open field arena and by spontaneous alternation (Y-maze) tests. **b** Number of buried marbles by 1-month-old female offspring of dams treated with vehicle versus IFNβ, during the first 5 min of the marble burying test (Student’s *t* test: *t*_(one tailed)_= 1.903, *df* = 12, *p = 0.0406). **c** Percent stranger exploration time by 3-month-old female offspring in social preference test (Student’s *t* test: *t*_(two tailed)_ = 2.446*, df* = 13, *p = 0.0294), and **d** representative heatmaps. STR -stranger mouse, OBJ -novel object. **e** Percent spontaneous alternation in Y-maze by 4-month-old female offspring (Student’s *t* test: *t*_(one tailed)_ = 1.808*, df* = 13, *p = 0.0469). **f** Time spent in open arms of the elevated plus maze by 3-month-old male offspring (Student’s *t* test: *t*_(two tailed)_ = 2.212*, df* = 13, *p = 0.0455), and **g** representative heatmaps. **h** Total distance of 3-month-old male offspring in the elevated plus maze arena. **i** Time spent in the center of the open field arena by 4-month-old male offspring (Student’s *t* test: *t*_(one tailed)_ = 1.410*, df* = 13*, p* = 0.091), and **j** total distance covered in the arena. Data are presented as means ± s.e.m. *n* = 5–10 offspring of 2–3 dams per group

The results above encouraged us to examine whether the prenatal exposure to IFNβ would have any effect on the behavior of the offspring at adolescence and adulthood (Fig. 4, Supplementary Fig. 1). For this purpose, pregnant dams were injected on E14.5 with vehicle or a high dose of IFNβ (45,400 U) to elicit a strong response, and behavior of the offspring was assessed. Specifically, at the age of 1 month we evaluated the repetitive behavior and anxiety of the offspring using marble burying and elevated plus maze tests [66, 67]. At the age of 3 months the offspring were tested for sociability by social preference test [68], and again by marble burying and elevated plus maze tests. At the age of 4 months the offspring were tested for anxiety by open field arena [69, 70] and for working memory by spontaneous alternation test in the Y-maze [71] (Fig. 4a). Overall, we found different response of the sexes to the maternal IFNβ treatment. In female offspring, we found at the age of 1 month that the IFNβ group buried a significantly higher number of marbles in the first 5 min of the marble burying test, as compared with the control group (Fig. 4b). At the age of 3 months, we found a significant reduction in social preference following maternal IFNβ treatment (Fig. 4c, d), and at 4 months we found a significantly higher percentage of spontaneous alternation, compared with the control group (Fig. 4e). In male offspring, we found at the age of 3 months, reduced exploration time of open arms in the elevated plus maze following maternal IFNβ treatment (Fig. 4f, g). This was not due to reduced distance covered by the animals (Fig. 4h). At the age of 4 months, we detected a trend toward reduced time spent at the center of the open field arena following the maternal treatment, which was not accompanied by change in total distance covered by the animals (Fig. 4i, j). These results demonstrate that maternal upregulation of IFNβ is manifested by changes in behavior of the offspring with different characteristics in females and males.

### Elevated IFNβ during pregnancy increases the vulnerability of the offspring and their microglia to postnatal stress

**Fig. 5:**
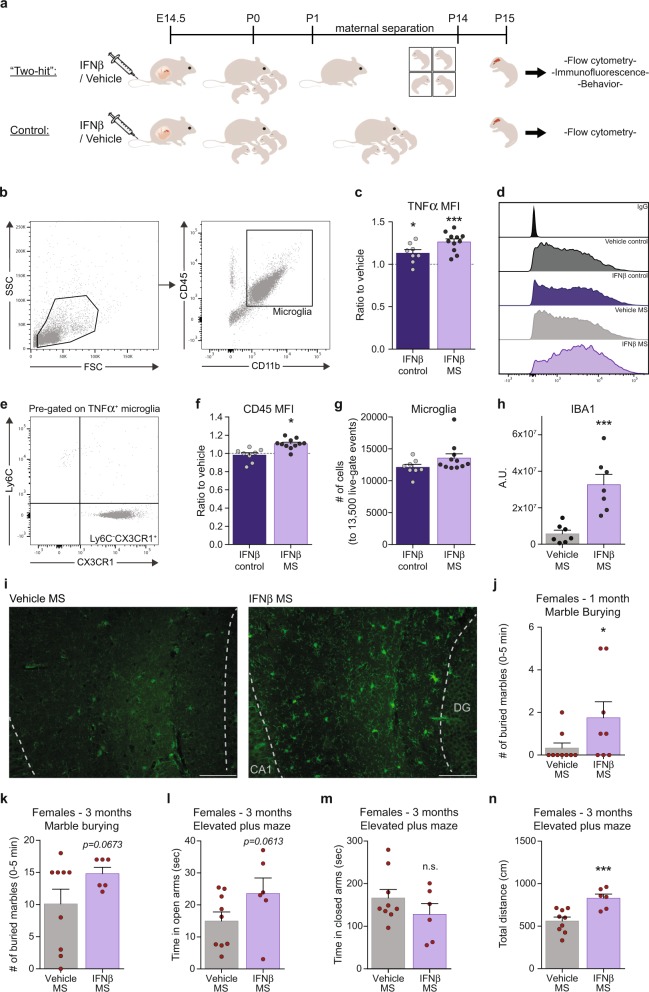
Maternal elevation of IFNβ increases the vulnerability of the offspring and their microglia to postnatal stress. **a** Pregnant females were i.v. injected on E14.5 with IFNβ (45,400 U) or vehicle. On postnatal days 1–14 (P1–P14), pups of the “two-hit” group were subjected to a 2-week maternal separation (MS) protocol. Pups of the control group were left with their mother. On P15, microglia were harvested from the pups, and were analyzed by flow cytometry and immunofluorescence. Offspring of the “two-hit” group were assessed at adolescence and adulthood with the same battery of behavioral tests, as described (Fig. 4a). **b** Flow cytometry gating strategy for offspring’s microglia. **c** Flow cytometry analysis showing the effect of maternal IFNβ treatment on microglial TNFα expression (mean fluorescence intensity; MFI) as the ratio relative to the relevant vehicle-treated group (control or MS) (Control groups—Student’s *t* test: *t*_(one tailed)_ = 1.968*, df* = 14, *p = 0.0346, Cohen’s *d* = 0.9838; MS groups—Student’s *t* test: *t*_(one tailed)_ = 5.018*, df* = 15, ***p = 0.0002, Cohen’s *d* = 2.71*)*. **d** Flow cytometry representative TNFα MFI of offspring’s microglia. **e** Representative flow cytometry dot plot for microglial Ly6C and CX3CR1 expression. **f** Flow cytometry analyses showing the effect of maternal IFNβ treatment on microglial CD45 expression as the ratio relative to the relevant vehicle-treated group (control or MS) (Student’s *t* test: *t*_(two tailed)_ = 2.578*, df* = 15, *p = 0.021), and **g** normalized number of microglia (to 13,500 live-gate events). **c**–**g** Representative results of one of two independent experiments. *n* = 6–11 offspring per group. **h** Immunofluorescence analysis and **i** representative images showing increased IBA-1 expression in hippocampi following maternal IFNβ treatment in the MS groups (Student’s *t* test: *t*_(two tailed)_ = 4.507*, df* = 12, ***p = 0.0007). DG -dentate gyrus, CA1 -Cornu Ammonis 1. *n* = 7 offspring per group. Scale bar, 100 µm. **j** Number of buried marbles during the first 5 min of the marble burying test by 1-month-old female offspring of the MS groups (Student’s *t* test: *t*_(one tailed)_ = 1.895*, df* = 15, *p = 0.0388). **k** Number of buried marbles during the first 5 min of the marble burying test by 3-month-old female offspring of the MS groups (Student’s *t* test: *t*_(one tailed)_ = 1.596*, df* = 13*, p* = 0.0673). **l** Time spent in open arms (Student’s *t* test: *t*_(one tailed)_ = 1.651*, df* = 13*, p* = 0.0613), **m** in closed arms and **n** total distance (Student’s *t* test: *t*_(one tailed)_ = 3.906*, df* = 13, ***p = 0.0009) covered by 3-month-old female offspring of the MS groups in the elevated plus maze. *n* = 6–9 offspring of 3 dams per group. **c**, **f–h**, **j–n** Data are presented as means ± s.e.m

Finally, we examined whether the exposure to the upregulated maternal IFNβ during pregnancy renders the offspring’s microglia less resilient to stressful conditions. We envisioned that this would be in line with the “Two-hit” model of neuropsychiatric disorders, according to which two “hits” are required for the pathology to occur; a first “hit” during prenatal life disrupts the offspring’s CNS development, thereby causes increased vulnerability to a second “hit”, which might occur later in life and would lead to the pathological onset of disease [19–22]. To this end, pregnant dams were injected on E14.5 with vehicle or a high dose of IFNβ (45,400 U) (first “hit”), and the newborn pups were subsequently subjected to a 2-week maternal separation (MS) protocol [on postnatal days 1–14 (P1–P14)] as a source of stress (second “hit”). Control groups were left with their mothers (Fig. 5a). We tested whether microglia of offspring that were subjected to MS would show a stronger inflammatory response if the dams were first injected with IFNβ during pregnancy. For this, microglia were harvested from the pups on P15, and were analyzed by flow cytometry (Fig. 5b–g) and by immunofluorescence (Fig. 5h, i) to detect changes in their inflammatory and activation state. We started by measuring microglial TNFα expression by flow cytometry. To analyze the entire microglial population, including activated cells, we analyzed all the CD11b^+^CD45^+^cells (Fig. 5b). Most of the cells were CD11b^int^CD45^int^, and thus mainly represented the microglial population. The effect of maternal IFNβ treatment on microglial TNFα expression was calculated as the expression ratio relative to the relevant vehicle-treated group (control or MS). In both control and MS groups, the microglial TNFα mean fluorescence intensity (MFI) was significantly increased in samples derived from pups of dams treated with IFNβ, relative to vehicle (Fig. 5c, d). Calculations of effect size using Cohen’s *d* showed an effect size of *d* = 0.9838 for the IFNβ treatment in the control groups, and an effect size of *d* = 2.71 for IFNβ treatment in the MS groups. To verify that the changes in TNFα occurred mainly in the microglia and were not due infiltration of monocyte-derived macrophages, expression of Ly6C and CX3CR1 was measured for the CD11b^+^CD45^+^TNFα^+^ cells (Fig. 5e). We found that, on average, 99.19 ± 0.037% of the cells were Ly6C^−^CX3CR1^+^, suggesting that TNFα was produced almost exclusively by microglia. In addition, we assessed microglial CD45 expression and found that following maternal treatment with IFNβ, the microglial CD45 MFI was higher only after MS, while expression in the control group remained unchanged (Fig. 5f). The results were not accompanied by any changes in the total number of microglia (Fig. 5g). Next, brains of the offspring from the MS groups (IFNβ and vehicle) were isolated, and analyzed using immunofluorescence to estimate microglial activation [86]. Analysis of IBA-1 immunoreactivity in hippocampi revealed increased expression in sections derived from brains of IFNβ–MS pups, as compared with the vehicle-MS group (Fig. 5h, i). These results showed that maternal elevation of IFNβ increased the sensitivity of the offspring-derived microglia to postnatal stress. Finally, we tested whether IFNβ exacerbates the effect of MS at the behavioral level. Offspring from the MS groups (IFNβ and vehicle) were examined with the same battery of tests as described above (Fig. 4a). Overall, we found different responses of the sexes to the treatment, with the main effect in females (Fig. 5j–n, Supplementary Fig. 2). At the age of 1 month, female offspring of the IFNβ–MS group buried a significantly higher number of marbles within the first 5 min of the test, relative to the vehicle-MS group (Fig. 5j). At the age of 3 months, IFNβ–MS female offspring showed a trend of burying a higher number of marbles, as compared with the vehicle-MS group (Fig. 5k). Those females also demonstrated a trend of spending more time in the open arms of the elevated plus maze (Fig. 5l), which may suggest increased risk-taking behavior, since they did not spend more time in closed arms, although they covered a significantly greater distance (Fig. 5m, n). These results demonstrate that maternal elevation of IFNβ renders the offspring less resilient to stressful conditions.

## Discussion

In this study, we found that MIA affects the offspring, at least in part, through a Type-I interferon-dependent response of the dam. Specifically, we found that MIA leads to a reduction in microglial proliferation in the newborn offspring. The absence of IFN-I signaling resulted in increased newborn microglial proliferation, while IFN-I blockage mitigated the detrimental effect on microglia elicited by the MIA. Moreover, systemic elevation of IFNβ in dams, in the absence of MIA, resulted in a decrease in microglial proliferation in the newborn pups, and increased the sensitivity of the microglia to postnatal stress. In addition, the maternal IFNβ treatment led to alterations in behavior of the offspring in adolescence and adulthood, and contributed to increased vulnerability of the offspring to stressful conditions.

MIA, induced by poly(I:C), is a well-established model, characterized by late onset behavioral deficits associated with autism and schizophrenia [1, 4, 8]. Although some studies found epigenetic changes in brains of the offspring [87, 88], the etiology and mechanisms forming the basis of this phenomenon are still unclear. Several studies examined changes in microglia of the pups following MIA by poly(I:C). In most cases, the activation state of microglia was assessed, but contradictory results have been reported; while some studies found increased microglial activation [55, 89–91], others failed to observe similar changes [47–49, 92, 93]. Here, in order to study early effects of MIA, we used the poly(I:C) model and focused on early time points following the maternal treatment. We found reduced microglial proliferation in the newborn pups as a result of the maternal challenge.

During development, microglia undergo distinct developmental stages associated with the changing microenvironment of the CNS, whereas different perturbations could lead to deviations in the maturation program [74]. Our results, demonstrating reduced newborn’s microglial proliferation following MIA, could indicate premature differentiation of the microglia and unsynchronized development relative to the rest of the CNS, which could manifest in neuropsychiatric pathologies.

One of the factors that could impair the developing embryo during maternal viral infection, is the maternal reaction to the virus. The antiviral response involves elevation of various inflammatory cytokines including IL-6, which was shown to induce endocrine changes in the placenta, leading to behavioral deficits in the offspring, and changes in gene-expression in their brains [11, 13, 14]. The antiviral maternal reaction also includes upregulation of IFN-I, the first line of defense against viral infections in mammals [17, 18, 94]. A recently published paper demonstrated that interferon receptor-1 (IFNAR) signaling, due to Zika virus infection during pregnancy, inhibits the development of the placenta and induces fetal resorption [95].

Here, we used maternal poly(I:C) administration, one of the common rodent models for MIA, that mimics viral infection and was shown to upregulate inflammatory cytokines, including IFN-I [4, 9–12, 15, 16]. Our results, 1 day after the MIA, demonstrated a IFN-I signature at the yolk sac, the origin of microglia progenitors [82, 83], and a subsequent regulatory effect of IFN-I on newborn’s microglia. Moreover, we showed that systemic elevation of IFNβ in dams was sufficient to impair the newborn’s microglial proliferation, and to increase the susceptibility of these cells to stress.

IFNβ was demonstrated to have a regulatory role in microglia; it was shown that microglia of mice deficient in IFN regulatory factor 7 (IRF7) or IFNAR exhibited increased expression of the C-X-C motif chemokine ligand 13 (CXCL13), in homeostasis, and to a greater extent, in response to viral encephalitis [96]. Moreover, our group previously showed that the microglial “phenotype-switch” in adulthood under injurious conditions is dependent on IFNβ signaling, and more specifically, on the transcription factor, IRF7 [39]. In addition, several studies described a negative effect of IFN-I/IFNβ on microglia in various cases, such as in systemic lupus erythematosus [97], in ageing [46], and when chronically elevated in adulthood [44, 45].

IFN-I signaling was demonstrated in the past as an important factor in arresting the reaction of myeloid cells to inflammatory stimuli, such as that mediated by IFNα, IFNγ, and lipopolysaccharide (LPS) [98]. It was also suggested that IFN-I might be important during development for the regulation of cell proliferation and/or differentiation [99], but this hypothesis has never been substantiated. Here, we found that newborn offspring that lack IFN-I signaling (IFNARKO mice) had a higher percentage of proliferating microglia, and in the case of MIA, this percentage was further increased. These results imply that IFN-I signaling is important for normal development of microglia. Yet, additional factors might be involved in regulation of microglial proliferation in homeostasis and following maternal poly(I:C) challenge, that are masked in the presence of IFN-I signaling [9–12]. Assessment of some of these factors, such as the Type-II interferon, IFNγ, and the NF-kB-dependent inflammatory cytokine, TNFα, revealed that they did not elicit the same unique effect on the newborn’s microglia following maternal injection, as induced by maternal elevation of IFNβ. These results could explain, to some extent, differences in findings elicited by MIA using the viral mimetic poly(I:C) versus the bacterial endotoxin LPS, a toll-like receptor 4 ligand [100]; LPS signaling induces a rapid production of NF-kB-dependent proinflammatory cytokines, while the production of IFN-I is delayed and in low levels [101].

IFN-I/IFNβ signaling was shown to affect several aspects of brain pathology and behavior. On the one hand, chronic IFN-I signaling in the ageing brain was demonstrated to negatively affect the choroid plexus and brain function [102]. On the other hand, treatment of IFNβ was found to be beneficial in cases of multiple sclerosis [56, 103, 104] and to exert a protective therapeutic effect in a mouse model of cerebral ischemia [105]. Furthermore, IFN-I/IFNβ was shown to play a role in brain homeostasis, specifically in neurons. Neurons were found to maintain a homeostatic IFN-I level that is optimal for early control of viral infection [106], while the lack of neuronal IFNβ signaling was shown to lead to defects in neuronal autophagy, to Lewy body accumulation, and to a Parkinson’s disease-like dementia [107]. Here, we found that maternal IFNβ treatment led to behavioral alterations in adolescent and adult offspring that were manifested in a sex-specific manner. Specifically, we demonstrated that female offspring show a higher degree of repetitive behavior alongside reduced sociability, and that male offspring exhibit increased anxiety, all of which are hallmark symptoms observed in neuropsychiatric disorders, such as in autism- and schizophrenia-spectrum disorders [108–114]. The improved performance of female offspring in the spontaneous alternation test may suggest enhanced working memory of these mice, which was also reported in autism spectrum disorder patients [115, 116]. Furthermore, we also demonstrated exacerbated behavioral changes following MS in female offspring of mothers that were injected with IFNβ; these changes were characterized by increased repetitive behavior and reduced anxiety/increased risk-taking behavior. Although those behavioral manifestations are not the same as those observed following maternal treatment with IFNβ alone, the different outcome may reflect a different spectrum-related neuropsychiatric disease, such as mania [7, 117].

Taken together, our results attribute a novel role to IFN-I in regulation of microglia during development, provided that its expression is well balanced; any deviation from the homeostatic level could affect their fate. Our findings further highlight that maternal upregulation of IFN-I, in response to viral infection, could be harmful to the developing offspring. Accordingly, monitoring maternal IFN-I during pregnancy, and specifically during the dam’s illness, would allow timely therapeutic intervention to modulate IFN-I levels, and could thereby help prevent such pathologies in the offspring.

## Supplementary information

Supplementary Figure Legends

Supplementary Figure 1

Supplementary Figure 2

Supplementary Table 1

Supplementary Table 2

Supplementary Table 3

## Data Availability

The data of this study are available from the corresponding author upon reasonable request.

## References

[1] Zuckerman L, Weiner I (2005). Maternal immune activation leads to behavioral and pharmacological changes in the adult offspring. J Psychiatr Res.

[2] Zuckerman L, Weiner I (2003). Post-pubertal emergence of disrupted latent inhibition following prenatal immune activation. Psychopharmacol.

[3] Li Q, Cheung C, Wei R, Hui ES, Feldon J, Meyer U (2009). Prenatal immune challenge is an environmental risk factor for brain and behavior change relevant to schizophrenia: evidence from MRI in a mouse model. PLoS ONE.

[4] Choi GB, Yim YS, Wong H, Kim S, Kim H, Kim SV (2016). The maternal interleukin-17a pathway in mice promotes autism-like phenotypes in offspring. Science.

[5] Brown AS (2006). Prenatal infection as a risk factor for schizophrenia. Schizophr Bull.

[6] Miller BJ, Culpepper N, Rapaport MH, Buckley P (2013). Prenatal inflammation and neurodevelopment in schizophrenia: a review of human studies. Prog Neuropsychopharmacol Biol Psychiatry.

[7] Tsuchiya KJ, Byrne M, Mortensen PB (2003). Risk factors in relation to an emergence of bipolar disorder: a systematic review. Bipolar Disord.

[8] Meyer U, Feldon J (2012). To poly(I:C) or not to poly(I:C): Advancing preclinical schizophrenia research through the use of prenatal immune activation models. Neuropharmacology.

[9] Murray C, Griffin ÉW, O’Loughlin E, Lyons A, Sherwin E, Ahmed S (2015). Interdependent and independent roles of type I interferons and IL-6 in innate immune, neuroinflammatory and sickness behaviour responses to systemic poly I: C. Brain Behav Immun.

[10] Meyer U, Feldon J, Yee BK (2009). A review of the fetal brain cytokine imbalance hypothesis of schizophrenia. Schizophr Bull.

[11] Smith SEP, Li J, Garbett K, Mirnics K, Patterson PH (2007). Maternal immune activation alters fetal brain development through interleukin-6. J Neurosci.

[12] Arrode-Brusés G, Brusés JL (2012). Maternal immune activation by poly I:C induces expression of cytokines IL-1β and IL-13, chemokine MCP-1 and colony stimulating factor VEGF in fetal mouse brain. J Neuroinflammation.

[13] Garbett Ka, Hsiao EY, Kálmán S, Patterson PH, Mirnics K (2012). Effects of maternal immune activation on gene expression patterns in the fetal brain. Transl Psychiatry.

[14] Hsiao EY, Patterson PH (2011). Activation of the maternal immune system induces endocrine changes in the placenta via IL-6. Brain Behav Immun.

[15] Alexopoulou L, Holt AC, Medzhitov R, Flavell RA (2001). Recognition of double-stranded RNA and activation of NF-kappaB by Toll-like receptor 3. Nature.

[16] Takeuchi O, Akira S (2007). Recognition of viruses by innate immunity. Immunol Rev.

[17] Schulz KS, Mossman KL (2016). Viral evasion strategies in Type I IFN signaling—a summary of recent developments. Front Immunol.

[18] García-Sastre A (2017). Ten strategies of interferon evasion by viruses. Cell Host Microbe.

[19] Feigenson KA, Kusnecov AW, Silverstein SM (2014). Inflammation and the two-hit hypothesis of schizophrenia. Neurosci Biobehav Rev.

[20] Monte AS, Mello BSF, Borella VCM, da Silva Araujo T, da Silva FER, Sousa FCFde (2017). Two-hit model of schizophrenia induced by neonatal immune activation and peripubertal stress in rats: study of sex differences and brain oxidative alterations. Behav Brain Res.

[21] Maynard TM, Sikich L, Lieberman JA, LaMantia AS (2001). Neural development, cell-cell signaling, and the ‘two-hit’ hypothesis of schizophrenia. Schizophr Bull.

[22] Giovanoli S, Engler H, Engler A, Richetto J, Voget M, Willi R (2013). Stress in puberty unmasks latent neuropathological consequences of prenatal immune activation in mice. Science.

[23] Heim C, Nemeroff CB (2001). The role of childhood trauma in the neurobiology of mood and anxiety disorders: preclinical and clinical studies. Biol Psychiatry.

[24] Tsuda MC, Ogawa S (2012). Long-lasting consequences of neonatal maternal separation on social behaviors in ovariectomized female mice. PLoS ONE.

[25] Michell-Robinson MA, Touil H, Healy LM, Owen DR, Durafourt BA, Bar-Or A (2015). Roles of microglia in brain development, tissue maintenance and repair. Brain.

[26] Nayak D, Roth TL, McGavern DB (2014). Microglia development and function. Annu Rev Immunol.

[27] Aguzzi A, Barres BA, Bennett ML (2013). Microglia: scapegoat, saboteur, or something else?. Science.

[28] Stevens B, Allen NJ, Vazquez LE, Howell GR, Christopherson KS, Nouri N (2007). The classical complement cascade mediates CNS synapse elimination. Cell.

[29] Schafer DP, Lehrman EK, Kautzman AG, Koyama R, Mardinly AR, Yamasaki R (2012). Microglia sculpt postnatal neural circuits in an activity and complement-dependent manner. Neuron.

[30] Paolicelli RC, Bolasco G, Pagani F, Maggi L, Scianni M, Panzanelli P (2011). Synaptic pruning by microglia is necessary for normal brain development. Science.

[31] Shigemoto-Mogami Y, Hoshikawa K, Goldman JE, Sekino Y, Sato K (2014). Microglia enhance neurogenesis and oligodendrogenesis in the early postnatal subventricular zone. J Neurosci.

[32] Cunningham CL, Martinez-Cerdeno V, Noctor SC (2013). Microglia regulate the number of neural precursor cells in the developing cerebral cortex. J Neurosci.

[33] Prinz M, Priller J (2014). Microglia and brain macrophages in the molecular age: from origin to neuropsychiatric disease. Nat Rev Neurosci.

[34] Rothhammer V, Borucki DM, Tjon EC, Takenaka MC, Chao C-C, Ardura-Fabregat A (2018). Microglial control of astrocytes in response to microbial metabolites. Nature.

[35] Kreutzberg GW (1996). Microglia: a sensor for pathological events in the CNS. Trends Neurosci.

[36] Pivneva TA (2008). Microglia in normal condition and pathology. Fiziol Zh.

[37] Deczkowska A, Amit I, Schwartz M (2018). Microglial immune checkpoint mechanisms. Nat Neurosci.

[38] Butovsky O, Jedrychowski MP, Moore CS, Cialic R, Lanser AJ, Gabriely G (2013). Identification of a unique TGF-β–dependent molecular and functional signature in microglia. Nat Neurosci.

[39] Cohen M, Matcovitch O, David E, Barnett-Itzhaki Z, Keren-Shaul H, Blecher-Gonen R (2014). Chronic exposure to TGFβ1 regulates myeloid cell inflammatory response in an IRF7-dependent manner. EMBO J.

[40] Honda K, Yanai H, Negishi H, Asagiri M, Sato M, Mizutani T (2005). IRF-7 is the master regulator of type-I interferon-dependent immune responses. Nature.

[41] Cronk JC, Derecki NC, Ji E, Xu Y, Lampano AE, Smirnov I (2015). Methyl-CpG binding protein 2 regulates microglia and macrophage gene expression in response to inflammatory stimuli. Immunity.

[42] Rossi C, Cusimano M, Zambito M, Finardi A, Capotondo A, Garcia-Manteiga JM (2018). Interleukin 4 modulates microglia homeostasis and attenuates the early slowly progressive phase of amyotrophic lateral sclerosis. Cell Death Dis.

[43] Lobo-Silva D, Carriche GM, Castro AG, Roque S, Saraiva M (2017). Interferon-β regulates the production of IL-10 by toll-like receptor-activated microglia. Glia.

[44] Goldmann T, Zeller N, Raasch J, Kierdorf K, Frenzel K, Ketscher L (2015). USP18 lack in microglia causes destructive interferonopathy of the mouse brain. EMBO J.

[45] Meuwissen MEC, Schot R, Buta S, Oudesluijs G, Tinschert S, Speer SD (2016). Human USP18 deficiency underlies type 1 interferonopathy leading to severe pseudo-TORCH syndrome. J Exp Med.

[46] Deczkowska A, Matcovitch-Natan O, Tsitsou-Kampeli A, Ben-Hamo S, Dvir-Szternfeld R, Spinrad A (2017). Mef2C restrains microglial inflammatory response and is lost in brain ageing in an IFN-I-dependent manner. Nat Commun.

[47] Smolders S, Smolders SMT, Swinnen N, Gärtner A, Rigo J-M, Legendre P (2015). Maternal immune activation evoked by polyinosinic:polycytidylic acid does not evoke microglial cell activation in the embryo. Front Cell Neurosci.

[48] Giovanoli S, Notter T, Richetto J, Labouesse MA, Vuillermot S, Riva MA (2015). Late prenatal immune activation causes hippocampal deficits in the absence of persistent inflammation across aging. J Neuroinflammation.

[49] Giovanoli S, Weber-Stadlbauer U, Schedlowski M, Meyer U, Engler H (2016). Prenatal immune activation causes hippocampal synaptic deficits in the absence of overt microglia anomalies. Brain Behav Immun.

[50] Patrich E, Piontkewitz Y, Peretz A, Weiner I, Attali B (2016). Maternal immune activation produces neonatal excitability defects in offspring hippocampal neurons from pregnant rats treated with poly I:C. Sci Rep.

[51] Zhang Z, van Praag H (2015). Maternal immune activation differentially impacts mature and adult-born hippocampal neurons in male mice. Brain Behav Immun.

[52] Coiro P, Padmashri R, Suresh A, Spartz E, Pendyala G, Chou S (2015). Impaired synaptic development in a maternal immune activation mouse model of neurodevelopmental disorders. Brain Behav Immun.

[53] Sekar A, Bialas AR, de Rivera H, Davis A, Hammond TR, Kamitaki N (2016). Schizophrenia risk from complex variation of complement component 4. Nature.

[54] Zhan Y, Paolicelli RC, Sforazzini F, Weinhard L, Bolasco G, Pagani F (2014). Deficient neuron-microglia signaling results in impaired functional brain connectivity and social behavior. Nat Neurosci.

[55] Eßlinger M, Wachholz S, Manitz M-P, Plümper J, Sommer R, Juckel G (2016). Schizophrenia associated sensory gating deficits develop after adolescent microglia activation. Brain Behav Immun.

[56] Prinz M, Schmidt H, Mildner A, Knobeloch K-P, Hanisch U-K, Raasch J (2008). Distinct and nonredundant in vivo functions of IFNAR on myeloid cells limit autoimmunity in the central nervous system. Immunity.

[57] Jung S, Aliberti J, Graemmel P, Sunshine MJ, Kreutzberg GW, Sher A (2000). Analysis of fractalkine receptor CX3CR1 function by targeted deletion and green fluorescent protein reporter gene insertion. Mol Cell Biol.

[58] Meyer U, Nyffeler M, Schwendener S, Knuesel I, Yee BK, Feldon J (2008). Relative prenatal and postnatal maternal contributions to schizophrenia-related neurochemical dysfunction after in utero immune challenge. Neuropsychopharmacology.

[59] Meyer U, Nyffeler M, Engler A, Urwyler A, Schedlowski M, Knuesel I (2006). The time of prenatal immune challenge determines the specificity of inflammation-mediated brain and behavioral pathology. J Neurosci.

[60] Sheehan KCF, Lai KS, Dunn GP, Bruce AT, Diamond MS, Heutel JD (2006). Blocking monoclonal antibodies specific for mouse IFN-alpha/beta receptor subunit 1 (IFNAR-1) from mice immunized by in vivo hydrodynamic transfection. J Interferon Cytokine Res.

[61] Belardelli F, Gabriele L, Proietti E, Sestili P, Peretti M, Rozera C (1991). Synergistic anti-tumor effects of combined IL-1/IFN-α/β therapy in mice injected with met astatic friend erythroleukemia cells. Int J Cancer.

[62] Liu H-Y, Liu Z-K, Chao H, Li Z, Song Z, Yang Y (2014). High-dose interferon-γ promotes abortion in mice by suppressing Treg and Th17 polarization. J Inter Cytokine Res.

[63] Clark IA, Chaudhri G (1988). Tumor necrosis factor in malaria-induced abortion. Am J Trop Med Hyg.

[64] Roque A, Ochoa-Zarzosa A, Torner L (2016). Maternal separation activates microglial cells and induces an inflammatory response in the hippocampus of male rat pups, independently of hypothalamic and peripheral cytokine levels. Brain Behav Immun.

[65] Deacon RMJ (2006). Digging and marble burying in mice: simple methods for in vivo identification of biological impacts. Nat Protoc.

[66] Thomas A, Burant A, Bui N, Graham D, Yuva-Paylor LA, Paylor R (2009). Marble burying reflects a repetitive and perseverative behavior more than novelty-induced anxiety. Psychopharmacology.

[67] Walf AA, Frye CA (2007). The use of the elevated plus maze as an assay of anxiety-related behavior in rodents. Nat Protoc.

[68] Moy S, Nadler J, Perez A, Barbaro RP, Johns JM, Magnuson TR (2004). Sociability and preference for social novelty in five inbred strains: an approach to assess autistic-like behavior in mice. Genes Brain Behav.

[69] Gould TD, Dao DT, Kovacsics CE (2009). The open field test. in mood and anxiety related phenotypes in mice. Neuromethods.

[70] Seibenhener ML, Wooten MC. Use of the open field maze to measure locomotor and anxiety-like behavior in mice. J Vis Exp. 2015;96:e52434. 10.3791/52434 PMC4354627 25742564

[71] Hughes RN (2004). The value of spontaneous alternation behavior (SAB) as a test of retention in pharmacological investigations of memory. Neurosci Biobehav Rev.

[72] Kouzu Y, Moriya T, Takeshima H, Yoshioka T, Shibata S (2000). Mutant mice lacking ryanodine receptor type 3 exhibit deficits of contextual fear conditioning and activation of calcium/calmodulin-dependent protein kinase II in the hippocampus. Mol Brain Res.

[73] Jaitin DA, Kenigsberg E, Keren-Shaul H, Elefant N, Paul F, Zaretsky I (2014). Massively parallel single-cell RNA-seq for marker-free decomposition of tissues into cell types. Science.

[74] Matcovitch-Natan O, Winter DR, Giladi A, Vargas Aguilar S, Spinrad A, Sarrazin S (2016). Microglia development follows a stepwise program to regulate brain homeostasis. Science.

[75] Trapnell C, Pachter L, Salzberg SL (2009). TopHat: discovering splice junctions with RNA-Seq. Bioinformatics.

[76] Heinz S, Benner C, Spann N, Bertolino E, Lin YC, Laslo P (2010). Simple combinations of lineage-determining transcription factors prime cis-regulatory elements required for macrophage and B cell identities. Mol Cell.

[77] Ashburner M, Ball CA, Blake JA, Botstein D, Butler H, Cherry JM (2000). Gene ontology: tool for the unification of biology. The gene ontology consortium. Nat Genet.

[78] Carbon S, Dietze H, Lewis SE, Mungall CJ, Munoz-Torres MC, Basu S (2017). Expansion of the gene ontology knowledgebase and resources: the gene ontology consortium. Nucleic Acids Res.

[79] Eden E, Lipson D, Yogev S, Yakhini Z (2007). Discovering motifs in ranked lists of DNA sequences. PLoS Comput Biol.

[80] Eden E, Navon R, Steinfeld I, Lipson D, Yakhini Z (2009). GOrilla: a tool for discovery and visualization of enriched GO terms in ranked gene lists. BMC Bioinform.

[81] Rusinova I, Forster S, Yu S, Kannan A, Masse M, Cumming H (2013). Interferome v2.0: an updated database of annotated interferon-regulated genes. Nucleic Acids Res.

[82] Ginhoux F, Greter M, Leboeuf M, Nandi S, See P, Gokhan S (2010). Fate mapping analysis reveals that adult microglia derive from primitive macrophages. Science.

[83] Schulz C, Gomez Perdiguero E, Chorro L, Szabo-Rogers H, Cagnard N, Kierdorf K (2012). A lineage of myeloid cells independent of Myb and hematopoietic stem cells. Science.

[84] Tak PP, Firestein GS (2001). NF-kappaB: a key role in inflammatory diseases. J Clin Investig.

[85] Greter M, Lelios I, Croxford AL (2015). Microglia versus myeloid cell nomenclature during brain inflammation. Front Immunol.

[86] Ito D, Tanaka K, Suzuki S, Dembo T, Fukuuchi Y (2001). Enhanced expression of Iba1, ionized calcium-binding adapter molecule 1, after transient focal cerebral ischemia in rat brain. Stroke.

[87] Basil P, Li Q, Dempster EL, Mill J, Sham P-C, Wong CCY (2014). Prenatal maternal immune activation causes epigenetic differences in adolescent mouse brain. Transl Psychiatry.

[88] Richetto J, Massart R, Weber-Stadlbauer U, Szyf M, Riva MA, Meyer U (2017). Genome-wide DNA methylation changes in a mouse model of infection-mediated neurodevelopmental disorders. Biol Psychiatry.

[89] Juckel G, Manitz MP, Brüne M, Friebe A, Heneka MT, Wolf RJ (2011). Microglial activation in a neuroinflammational animal model of schizophrenia—a pilot study. Schizophr Res.

[90] Manitz MP, Plümper J, Demir S, Ahrens M, Eßlinger M, Wachholz S (2016). Flow cytometric characterization of microglia in the offspring of polyI:C treated mice. Brain Res.

[91] Mattei D, Ivanov A, Ferrai C, Jordan P, Guneykaya D, Buonfiglioli A (2017). Maternal immune activation results in complex microglial transcriptome signature in the adult offspring that is reversed by minocycline treatment. Transl Psychiatry.

[92] Garay Pa, Hsiao EY, Patterson PH, McAllister a. K (2013). Maternal immune activation causes age- and region-specific changes in brain cytokines in offspring throughout development. Brain Behav Immun.

[93] Willi R, Harmeier A, Giovanoli S, Meyer U (2013). Altered GSK3β signaling in an infection-based mouse model of developmental neuropsychiatric disease. Neuropharmacology.

[94] Haller O, Kochs G, Weber F (2006). The interferon response circuit: Induction and suppression by pathogenic viruses. Virology.

[95] Yockey LJ, Jurado KA, Arora N, Millet A, Rakib T, Milano KM (2018). Type I interferons instigate fetal demise after Zika virus infection. Sci Immunol.

[96] Esen N, Rainey-Barger EK, Huber AK, Blakely PK, Irani DN (2014). Type-I interferons suppress microglial production of the lymphoid chemokine, CXCL13. Glia.

[97] Bialas AR, Presumey J, Das A, van der Poel CE, Lapchak PH, Mesin L (2017). Microglia-dependent synapse loss in type I interferon-mediated lupus. Nature.

[98] Hwang SY, Hertzog PJ, Holland KA, Sumarsono SH, Tymms MJ, Hamilton JA (1995). A null mutation in the gene encoding a type I interferon receptor component eliminates antiproliferative and antiviral responses to interferons alpha and beta and alters macrophage responses. Proc Natl Acad Sci USA.

[99] Hertzog P J, Hwang S Y, Kola I (1994). Role of interferons in the regulation of cell proliferation, differentiation, and development. Mol Reprod Dev.

[100] Meyer U (2014). Prenatal poly(I:C) exposure and other developmental immune activation models in rodent systems. Biol Psychiatry.

[101] Pålsson-McDermott EM, O’Neill LAJ (2004). Signal transduction by the lipopolysaccharide receptor, Toll-like receptor-4. Immunology.

[102] Baruch K, Deczkowska A, David E, Castellano JM, Miller O, Kertser A (2014). Aging-induced type I interferon response at the choroid plexus negatively affects brain function. Science.

[103] Río J, Tintoré M, Nos C, Téllez N, Galán I, Montalban X (2005). Interferon beta in relapsing–remitting multiple sclerosis. J Neurol.

[104] Group TIMSS. (1993). Interferon beta-1b is effective in relapsing-remitting multiple sclerosis. I. clinical results of a multicenter, randomized, double-blind, placebo-controlled trial. The IFNB multiple sclerosis study group. Neurology.

[105] Kuo P-C, Scofield BA, Yu I-C, Chang F-L, Ganea D, Yen J-H (2016). Interferon-β modulates inflammatory response in cerebral ischemia. J Am Heart Assoc.

[106] Cavanaugh SE, Holmgren AM, Rall GF (2015). Homeostatic interferon expression in neurons is sufficient for early control of viral infection. J Neuroimmunol.

[107] Ejlerskov P, Hultberg JG, Wang J, Carlsson R, Ambjørn M, Kuss M (2015). Lack of neuronal IFN-β-IFNAR causes Lewy body- and Parkinson’s disease-like dementia. Cell.

[108] Lord C, Elsabbagh M, Baird G, Veenstra-Vanderweele J (2018). Autism spectrum disorder. Lancet.

[109] Hommer RE, Swedo SE (2015). Schizophrenia and autism-related disorders. Schizophr Bull.

[110] Gaebel W, Barch DM, Bustillo J, Gur RE, Heckers S, Malaspina D (2013). Definition and description of schizophrenia in the DSM-5. Schizophr Res.

[111] Owen MJ, Sawa A, Mortensen PB (2016). Schizophrenia. Lancet.

[112] Temmingh H, Stein DJ (2015). Anxiety in patients with schizophrenia: epidemiology and management. CNS Drugs.

[113] American Psychiatric Association. Neurodevelopmental disorders. In: Diagnostic and statistical manual of mental disorders (5th ed.). American Psychiatric Association; Arlington, 2013. 10.1176/appi.books.9780890425596.dsm01.

[114] American Psychiatric Association. Schizophrenia spectrum and other psychotic disorders. In: Diagnostic and statistical manual of mental disorders (5th ed.). American Psychiatric Association; Arlington, 2013. 10.1176/appi.books.9780890425596.dsm02.

[115] Edgin JO, Pennington BF (2005). Spatial cognition in autism spectrum disorders: superior, impaired, or just intact?. J Autism Dev Disord.

[116] Beversdorf DQ, Smith BW, Crucian GP, Anderson JM, Keillor JM, Barrett AM (2000). Increased discrimination of ‘false memories’ in autism spectrum disorder. Proc Natl Acad Sci USA.

[117] Miró X, Meier S, Dreisow ML, Frank J, Strohmaier J, Breuer R (2012). Studies in humans and mice implicate neurocan in the etiology of mania. Am J Psychiatry.

