# Supplementary material for: Maternal Type-I interferon signaling adversely affects the microglia and the behavior of the offspring accompanied by increased sensitivity to stress
Source: Mol Psychiatry. 2019 Nov 26;25(5):1050–67. doi: 10.1038/s41380-019-0604-0 (PMC7192855; doi:10.1038/s41380-019-0604-0)
Supplement: Supplementary file 1 — Supplementary Figure Legends [file 41380_2019_604_MOESM1_ESM.docx]

**Supplementary Figure 1. Normal behavioral parameters of offspring following maternal IFNβ treatment. a,** Time spent in open arms and **b,** total distance (Student’s *t*-test: *t_(one-tailed)_=1.783, df=13, *p=0.0489*) covered by 1 month old female offspring of dams treated with vehicle or IFNβ in the elevated plus maze. **c,** Time spent in open arms and **d,** total distance covered by 3 month old female offspring in the elevated plus maze. **e,** Number of buried marbles during the first 5 minutes of the marble burying test by 3 month old female offspring. **f,** Time spent in the center of the open field arena by 4 month old females and **g,** total distance covered in the arena. **h,** Time spent in open arms and **i,** total distance covered by 1 month old male offspring to dams treated with vehicle or IFNβ in the elevated plus maze. **j,** Number of buried marbles during the first 5 minutes of the marble burying test by 1- and **k,** 3- month old male offspring. **l,** Percent stranger exploration time by 3 month old male offspring in social preference test. **m,** Percent spontaneous alternation in Y-maze by 4 month old male offspring. Data are presented as means ± s.e.m. N=6-10 offspring of 2-3 dams per group.

**Supplementary Figure 2. Behavioral parameters of offspring following a “two-hit” paradigm. a,** Time spent in open arms of the elevated plus maze and **b,** total distance covered in the maze by 1 month old female offspring that underwent maternal separation (MS) and whose mothers were treated during pregnancy with vehicle or IFNβ. **c,** Percent stranger exploration time by 3 month old female offspring in social preference test. **d,** Percent spontaneous alternation in Y-maze by 4 month old female offspring. **e,** Time spent in the center of the open field arena by 4 month old female and **f,** total distance covered in the arena. **g,** Time spent in open arms of the elevated plus maze and **h,** total distance (Student’s *t*-test: *t_(one-tailed)_=1.732, df=19, *p=0.0497*) covered in the maze by 1 month old male offspring that underwent maternal separation (MS) and whose mothers were treated during pregnancy with vehicle or IFNβ. **i,** Time spent in open arms of the elevated plus maze and **j,** total distance covered in the maze by 3 month old male offspring. **k,** Number of buried marbles during the first 5 minutes of the marble burying test by 1- and **l,** 3- month old male offspring. **m,** Percent stranger exploration time by 3 month old male offspring in social preference test. **n,** Percent spontaneous alternation in Y-maze by 4 month old male offspring. **o,** Time spent in the center of the open field arena by 4 month old male and **p,** total distance covered in the arena (Student’s *t*-test: *t_(one-tailed)_=2.096, df=17, *p=0.0257*). Data are presented as means ± s.e.m. N=6-9 offspring of 3 dams per group.
